# Dexamethasone uptake in the murine organ of Corti with transtympanic versus systemic administration

**DOI:** 10.1186/1916-0216-42-19

**Published:** 2013-02-27

**Authors:** Amandeep S Grewal, Julian M Nedzelski, Joseph M Chen, Vincent YW Lin

**Affiliations:** 1 Department of Otolaryngology-Head and Neck Surgery, Sunnybrook Health Sciences Centre, University of Toronto, 2075 Bayview Ave, Suite M1-102, Toronto, ON M4N 3M5, Canada

## Abstract

**Objective:**

To investigate glucocorticoid uptake in auditory hair cells following transtympanic versus systemic administration of dexamethasone.

**Study design:**

Controlled experimental study.

**Setting:**

Translational science experimental laboratory.

**Methods:**

Swiss-Webster mice were injected with dexamethasone via transtympanic or systemic administration. At 1, 6, or 12 hours post-injection the temporal bones were harvested. After cryosectioning, immunohistochemical staining was performed using an antibody for dexamethasone.

**Results:**

Dexamethasone labelling was greatest at 1 hour. Inner hair cells demonstrated much higher steroid uptake than outer hair cells. Both transtympanic injection and high-dose systemic administration resulted in strong dexamethasone labelling of hair cells, and a decreasing basal-to-apical gradient of hair cell fluorescence intensity was observed. Systemically delivered dexamethasone was rapidly eliminated from the inner ear, demonstrating mild labelling after 6 hours and none after 12 hours. In contrast, the mice receiving transtympanic injection had persistent moderate intensity fluorescence at 6 and 12 hours post-injection.

**Conclusion:**

There is similar uptake of dexamethasone by auditory hair cells after transtympanic and high-dose systemic delivery. Novel findings include the presence of a decreasing basal-apical gradient of steroid uptake, and demonstration of greater affinity of inner hair cells for dexamethasone compared to outer hair cells. In this animal model transtympanic injection resulted in prolonged steroid uptake. These findings help further our understanding of the pharmacokinetics of steroids in the cochlea, with a focus on auditory hair cells.

## Introduction

The utility of glucocorticoids in various otologic conditions is the focus of much recent and ongoing research. The ability to transtympanically deliver steroids to the middle ear is currently practiced in the treatment of inner ear disorders such as idiopathic sudden sensorineural hearing loss, Meniere’s disease, tinnitus, and autoimmune inner ear disease [1]–[7]. There is also an emerging otoprotective role for transtympanic steroids in cochlear implantation, acoustic trauma, and with ototoxic chemotherapeutics [8]–[13]. While systemic administration of glucocorticoids has been routine practice by otologists, potential toxicities are a limiting factor to long-term use. Side effects may include weight gain, cushingoid state, hypertension, osteoporosis, cataract formation, immunosuppression, psychosis, and avascular necrosis. Combined with the presence of a significant chemical blood-labyrinth barrier, these factors have motivated a shift towards injection of steroids directly into the middle ear in attempt to achieve high intra-cochlear drug levels. In order to design and optimize clinical therapies however, a detailed understanding of the pharmacokinetics of steroids in the inner ear is necessary.

Animal and human studies have demonstrated that glucocorticoids injected into the middle ear will permeate the round window membrane (RWM), and may achieve greater perilymph concentrations than by systemic administration [14]–[17]. Initial studies relied on perilymph sampling from the basal scala tympani, which is limited by potential CSF contamination via the cochlear aquaduct once the cochlear bony wall or RWM is breached [14]–[16]. Subsequent experiments using perilymph sampling from the cochlear apex demonstrated even higher glucocorticoid levels, as well as the presence of a decreasing basal-apical concentration gradient along the scala tympani [18]. Several studies have demonstrated peak perilymph glucocorticoid concentrations approximately 1 hour after RWM application or systemic delivery, with elimination of steroid from the perilymph approaching 6 hours [14,16,17,19].

The pharmacokinetics of the glucocorticoid dexamethasone in perilymph is well characterized, but the uptake of this compound into the Organ of Corti and its subsequent downstream effects with regards to upregulation of transcription factors is still poorly understood. The details on the actual cellular uptake are crucial to fully understand because it is directly related to the potential positive effects of glucocorticoids. An earlier immunohistochemical study demonstrated uniform dexamethasone uptake throughout the entire mouse cochlea after transtympanic administration including the organ of Corti, which is challenging to explain if a significant perilymph gradient exists [20]. Also, whether the elimination rate of glucocorticoids from auditory hair cells is similar to the perilymph is not known, and this has significant implications for designing therapeutic uses for these drugs.

The purpose of this study was to investigate the uptake and distribution of dexamethasone in murine auditory hair cells after transtympanic versus systemic administration. Of particular interest was the concentration of dexamethasone uptake and the time course of elimination.

## Materials and methods

### Mice

A total of 39 healthy adult Swiss-Webster mice were used to study the pharmacokinetics of dexamethasone within the organ of Corti. Animals were between 4 to 6 weeks of age and of approximately 25 grams bodyweight (Charles River Laboratories, Montreal QC, Canada). We obtained full approval for this study from the Sunnybrook Animal Care Committee.

### Dexamethasone injections

Dexamethasone sodium phosphate (Sandoz, Boucherville QC, Canada) was utilized in a concentration of 10 mg/ml, one of our standard formulations for patient use.

Swiss-Webster mice were anaesthetized using a custom inhalational anaesthetic chamber with 2.5% isoflurane in 2L/min oxygen.

Systemic administration: Once anaesthetized, hypodermic injection of dexamethasone solution in concentrations of 10 mg/kg of bodyweight (0.025 ml) or 100 mg/kg (0.25 ml) was delivered to the intraperitoneal cavity. Control animals were injected with normal saline. Anaesthesia was reversed and the animals were allowed to fully recover under a warming lamp before transfer back to their cages.

Transtympanic administration: Once anaesthetized, animals were removed from the inhalational chamber and placed in a standard murine nose cone providing the same anaesthetic. The right tympanic membrane was visualized using a surgical stereomicroscope. The opposite ear was left uninjected. A small myringotomy was performed in the superior aspect of the TM to allow air evacuation from the middle ear cavity during injection. Using a 27-gauge pediatric spinal needle, which was modified to a blunt tip, dexamethasone was gently injected through the tympanic membrane. An air-fluid meniscus was easily observed, and steroid was injected until the middle ear was entirely full. Mice were then placed in the anaesthetic chamber for 30 minutes with the injected ear up, followed by otoscopic confirmation of the persistence of dexamethasone solution in the middle ear cavity. Control animals were injected with normal saline. Anaesthesia was then reversed and the animals were allowed to fully recover under a warming lamp before transfer back to their cages.

### Immunohistochemical staining

Mice were anaesthetized with isoflurane and sacrificed by cervical dislocation at survival times of 1, 6, and 12 hours post-injection of transtympanic or systemic dexamethasone. The inner ears were quickly dissected free from the skull and middle ear bulla and immersed in ice-cold 1% fetal bovine serum in Dulbecco’s modified eagle medium solution. Under microscopy the stapes was removed and small otic capsule perforations performed at the cochlear apex and the superior semicircular canal. Samples were placed in 4% paraformaldehyde for 20 minutes, then decalcified in 10% ethylenediamine tetra-acetic acid for 24 hours at 4°C. Inner ears were transitioned through a gradient of sucrose (30%), half optimal cutting temperature compound (OCT) in sucrose, and full OCT for 24 hours in each of these solutions. Tissues were then embedded in OCT over dry ice and cryostat sectioned into 10μm slices and placed on glass slides for immunohistochemistry.

Dexamethasone primary antibodies (ABCAM, Cambridge MA, USA) were diluted to 1:250 in 20% normal goat serum (NGS) with 0.05% triton-X and were applied to the sectioned tissues and incubated overnight at 4°C. The secondary antibodies (Jackson Immunoresearch Laboratories, West Grove PA, USA) were diluted to 1:400 in 20% NGS with 0.05% triton-X and incubated at room temperature for 4 hours. Slides were then rinsed and co-labeled with phalloidin (Sigma-Aldrich, Oakville ON, Canada) before being mounted with Vectashield (Vector Laboratories, Burlington ON, Canada) for imaging. Untreated ears that served as the negative controls underwent the same immunohistochemical evaluation.

All sectioned tissues were reviewed and those demonstrating appropriate orientation of cellular anatomy and minimal fragmentation were selected for comparison. High-resolution imaging was performed using a Zeiss LSM510 confocal scanning system (Carl Zeiss MicroImaging GmbH, Germany) equipped with a Spectra Physics multi-line argon laser (Spectra Physics, Santa Clara CA, USA). Confocal and laser settings were kept consistent throughout all imaging analysis to minimize variability between experiments and samples.

Using high-definition monitors, two independent reviewers (ASG and VYWL) subjectively assessed the relative intensity of labeling for dexamethasone between the selected samples. Consistently observed patterns and differences were recorded, then discussed between reviewers and confirmed through repeat analysis.

## Results

**Figure 1:**
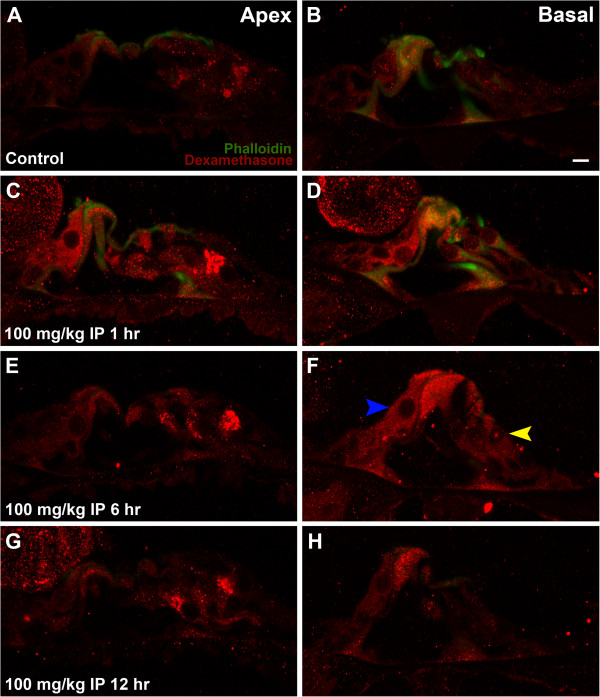
**(A) Apical region of control mouse; (B) Basal region of control mouse; (C) Apical region of extreme high dose positive control mouse at 1 hour post-injection; (D) Basal region of positive control mouse at 1 hour; (E) Apical region of positive control mouse at 6 hours; (F) Basal region of positive control mouse at 6 hours; (G) Apical region of positive control mouse at 12 hours; (H) Basal region of positive control mouse at 12 hours. **Blue arrow in Panel F marks inner hair cell and yellow arrow marks outer hair cells. White bar in Panel **B** represents 10 μm.

Minimal background fluorescence was observed in negative controls (Figure 1A-B). Positive control mice receiving extreme high dose systemic dexamethasone (100 mg/kg) yielded very strong intensity of hair cell labelling of the cochlear base and apex at 1 hour (Figure 1C-D). At 6 hours a decreasing basal-apical gradient becomes apparent, with moderate to strong labelling of hair cells at the base but minimal at the apex (Figure 1E-F). Minimal appreciable signal at 12 hours suggests near-complete elimination of dexamethasone by this time in the positive control mice (Figure 1G-H).

**Figure 2:**
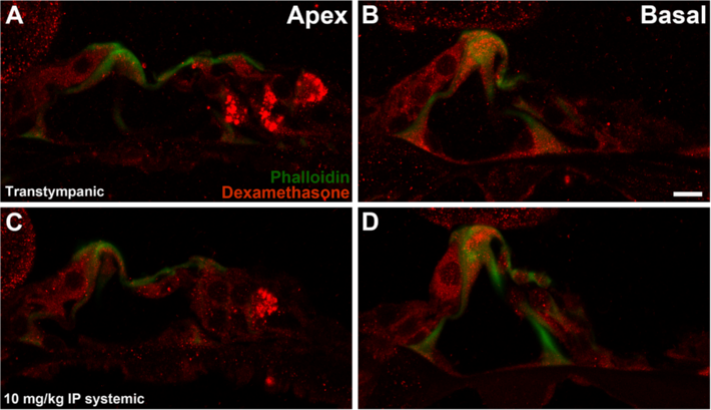
**(A) Apical region of transtympanic mouse at 1 hour post-injection; (B) Basal region of transtympanic mouse at 1 hour; (C) Apical region of high dose systemic mouse at 1 hour post-injection; (D) Basal region of systemic mouse at 1 hour. **White bar in Panel **B **represents 10 μm.

Both transtympanic and high-dose systemic (10 mg/kg) dosing demonstrated similar profiles of dexamethasone uptake by auditory hair cells at 1 hour (Figure 2). Hair cells in the basal organ of Corti demonstrate similar moderate to strong fluorescence in both groups (Figure 2B, 2D), though noticeably less than in the extreme high dose positive controls (Figure 1C). Labelling is substantially diminished at the cochlear apex in both groups (Figure 2A, 2C), suggesting the presence of a decreasing basal-apical gradient for dexamethasone uptake by auditory hair cells.

**Figure 3:**
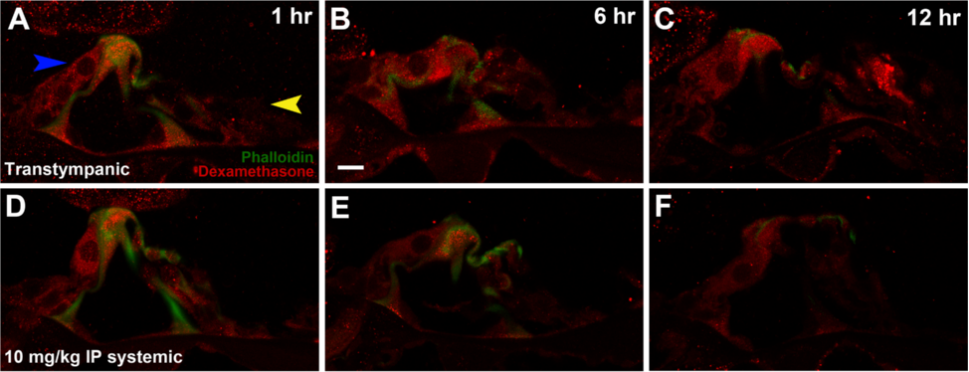
**(A) Basal region of transtympanic mouse at 1 hour post-injection; (B) Basal region of transtympanic mouse at 6 hours; (C) Basal region of transtympanic mouse at 12 hours; (D) Basal region of high dose systemic mouse at 1 hour post-injection; (E) Basal region of systemic mouse at 6 hours; (F) Basal region of systemic mouse at 12 hours. **Blue arrow in Panel **A** marks Inner hair cell and yellow arrow marks outer hair cells. White bar in Panel **B **represents 10 μm.

There was significantly greater uptake of dexamethasone by inner hair cells compared to outer hair cells. This difference was consistent for transtympanic and systemic dexamethasone administration (Figure 3A; blue arrow marking inner hair cell, Figure 3D). As such, the intensity of fluorescence of inner hair cells was primarily utilized to compare dexamethasone uptake patterns between groups.

Between transtympanic versus high-dose systemic administration, very different patterns of dexamethasone elimination from auditory hair cells were observed. The 1 hour fluorescent labelling of basal hair cells appeared equal between both groups (Figure 3A, 3D) and was moderate to strong in intensity. At 6 hours there was weak residual labelling after systemic delivery (Figure 3E), and at 12 hours there was no appreciable fluorescence (Figure 3F). In contrast, transtympanic delivery demonstrated moderately intense fluorescence at 6 and 12 hours (Figure 3B-C), suggesting a prolonged steroid effect after one-time transtympanic dexamethasone injection in this animal model. Preferential inner hair cell uptake was again observed (Figure 3A, blue arrow marks inner hair cell).

## Discussion

This study used immunohistochemical analysis to investigate dexamethasone uptake by auditory hair cells after transtympanic versus systemic administration. The utility of this modality to study hair cell steroid uptake is demonstrated in Figure 1, with minimal background labelling in negative controls (Figure 1A-B) and very strong labelling throughout the cochlea for positive control animals receiving extreme high dose dexamethasone (Figure 1C-D).

The preferential uptake of dexamethasone by inner hair cells compared to outer hair cells is striking, and to our knowledge not previously reported. Clear examples are demonstrated in Figures 1F and 3A (blue arrow marking inner hair cell and yellow arrow marking outer hair cells). This phenomenon was consistently observed for both transtympanic and systemic administration. Previous studies evaluating the oto-protective effects of glucocorticoids in acoustic trauma and aminoglycoside ototoxicity have clearly demonstrated greater sensitivity of outer hair cells to these cochlear insults, while inner hair cells are much more resistant [9,10,21]. With glucocorticoid administration inner hair cells were entirely preserved, while significantly more outer hair cells were preserved in a dose-dependent fashion. A greater affinity for glucocorticoids combined with patterns of endogenous steroid production may help explain the innate resistance of inner hair cells to ototoxic insults previously described.

In the present study both transtympanic and high dose systemic dexamethasone achieved strong uptake in basal inner hair cells at 1 hour, as demonstrated in Figure 2A and C. In contrast, Parnes et al. showed 1 hour dexamethasone peak levels in perilymph of 0.22 mg/L after high-dose intravenous (IV) injection, versus 1.553 mg/L in perilymph and 9.062 mg/L in endolymph after transtympanic injection [14]. Yang et al. found even higher perilymph concentrations at 1 hour of 2.17 mg/L after transtympanic injection [16]. Based on these previous studies of perilymph distribution it may be expected that hair cell uptake should be much lower with systemic versus transtympanic delivery. It was surprising to discover equally strong uptake of dexamethasone by auditory hair cells between the two delivery modalities.

After systemic and transtympanic injection, a decreasing basal-apical gradient of steroid uptake was present within hair cells which has not been previously characterized (Figure 2A-D). Salt et al. have demonstrated a perilymphatic gradient by sampling from the cochlear apex after transtympanic dexamethasone injection, generated by diffusion of steroid across the RWM [18]. Knowing this, it is intriguing to see that vascular delivery of dexamethasone to the cochlea after systemic administration also leads to a basal-apical gradient within auditory hair cells. Such a gradient may have significant implications for steroid efficacy in different regions of the cochlea. Although the only previous study that used immunohistochemistry to study cochlear dexamethasone uptake failed to demonstrate this phenomenon, findings from the present study appear to support the current understanding of cochlear dexamethasone pharmacokinetics [18,20].

Understanding the time course of steroid uptake and elimination from the cochlea is critical to the treatment of inner ear diseases. Previous studies have demonstrated peak perilymphatic concentrations at 30 min to 1 hour for systemic and transtympanic glucocorticoids [14,16,17,19,20]. At 6 hours, concentrations are markedly decreased, and at 24 hours glucocorticoids are largely eliminated from the inner ear. Building upon these data, this study evaluated hair cell dexamethasone uptake at 1, 6, and 12 hours. Temporal trends for high dose systemic administration demonstrated strong labelling at 1 hour, markedly decreased labelling at 6 hours and essentially no signal at 12 hours (Figure 3D-F), which parallels the aforementioned perilymph pharmacokinetics studies. However for transtympanic dexamethasone a very interesting effect of maximal 1 hour fluorescence (Figure 3A) and prolonged labelling of moderate strength up to 12 hours was observed (Figure 3B, 3C), suggesting retention of dexamethasone solution in the middle ear, continued permeation through the RWM into the perilymph, and continued auditory hair cell uptake. Eustachian tube losses and variable RWM permeability are challenges with transtympanic injection, but the present study demonstrates the potential for such strategies to markedly prolong glucocorticoid uptake by auditory hair cells. Preliminary work on sustained-release drug delivery vehicles such as dexamethasone hydrogels appears to demonstrate promising results [22,23].

Regarding study limitations, immunohistochemical analysis has not been traditionally utilized for precise quantification. However, more powerful and sensitive confocal microscopes along with better secondary antibodies have allowed the detection of even trace amounts of a molecule of interest, and as such the dynamic range of the fluorescent label as it relates to concentration has been more accepted. In this study, outcomes were measured by subjective assessment of labeling by two independent reviewers, limiting the results to a descriptive analysis and thereby avoiding any errors that may be introduced by attempting to develop a new objective assessment method. In future experiments, as steroid dosing is decreased closer to clinical levels, more subtle differences in labeling may require increased precision and necessitate developing a form of objective measurement. However the large doses and subsequent obvious differences in labeling between groups allowed us to easily identify patterns through observation alone. Importantly, to limit variability a rigorous methodology was adhered to for all experiments, control mice were utilized, and importantly the confocal and laser settings were kept identical throughout the study. All sections were processed using the same batch of reagents and antibodies, and using the same time and temperature parameters.

Systemic dexamethasone concentrations used in this study are greater than those used in clinical practice. Parnes et al. demonstrated that with IV dosing of 0.2 mg/kg of dexamethasone, based on the dosing used clinically in humans, there was no demonstrable uptake in the plasma, CSF or perilymph in guinea pigs. High dose IV dexamethasone at 8 mg/kg yielded 1 hour drug peaks in the perilymph of 0.22 mg/L and the plasma of 2.123 mg/L [14]. The dosing used in this study is in keeping with previously published literature. An extreme high dose of 100 mg/kg was also used as a maximal positive control (Figure 1), which would be toxic for clinical applications. This achieved very high intensity of labelling within hair cells, and interestingly this abolished the basal-apical gradient observed in the other experimental groups. Establishing that the organ of Corti can be maximally ‘saturated’ by such high dose dexamethasone, and that the basal-apical gradient can be overcome, serves as an intriguing metric with which to compare novel drug delivery strategies.

## Conclusion

This study demonstrated similar uptake of dexamethasone by auditory hair cells after transtympanic and high-dose systemic delivery. A decreasing basal-apical gradient of glucocorticoid uptake was observed in both groups, and while systemic administration resulted in quick elimination of steroid from hair cells, transtympanic injection lead to a prolonged effect. Interestingly the inner hair cells exhibit much higher affinity for dexamethasone than outer hair cells, though whether this phenomenon contributes to the greater resilience of inner hair cells to cochlear insult remains to be proven.

## Competing interests

The authors declare that they have no competing interests.

## Authors’ contributions

ASG carried out the animal experiments, performed confocal microscopy and analysis of results, and drafted the manuscript. JMN was involved in study design and drafting of the manuscript. JMC was involved in study design and drafting of the manuscript. VYWL was involved in study design, carried out the animal experiments, was involved in analysis of results, and drafted the manuscript. All authors read and approved the final manuscript.

## Sources of funding

Canadian Institutes of Health Research (CIHR)

Hearing Foundation of Canada (THFC)

## 

This material has never been published and is not currently under evaluation in any other peer-reviewed publication
